# Cluster Detection Mechanisms for Syndromic Surveillance Systems: Systematic Review and Framework Development

**DOI:** 10.2196/11512

**Published:** 2020-05-26

**Authors:** Prosper Kandabongee Yeng, Ashenafi Zebene Woldaregay, Terje Solvoll, Gunnar Hartvigsen

**Affiliations:** 1 Department of Computer Science University of Tromsø, The Arctic University of Norway Gjøvik Norway; 2 Department of Information Security and Communication Technology Norwegian University of Science and Technology Gjøvik Norway; 3 Norwegian Centre for E-health Research University Hospital Tromsø Norway

**Keywords:** sentinel surveillance, space-time clustering, aberration detection

## Abstract

**Background:**

The time lag in detecting disease outbreaks remains a threat to global health security. The advancement of technology has made health-related data and other indicator activities easily accessible for syndromic surveillance of various datasets. At the heart of disease surveillance lies the clustering algorithm, which groups data with similar characteristics (spatial, temporal, or both) to uncover significant disease outbreak. Despite these developments, there is a lack of updated reviews of trends and modelling options in cluster detection algorithms.

**Objective:**

Our purpose was to systematically review practically implemented disease surveillance clustering algorithms relating to temporal, spatial, and spatiotemporal clustering mechanisms for their usage and performance efficacies, and to develop an efficient cluster detection mechanism framework.

**Methods:**

We conducted a systematic review exploring Google Scholar, ScienceDirect, PubMed, IEEE Xplore, ACM Digital Library, and Scopus. Between January and March 2018, we conducted the literature search for articles published to date in English in peer-reviewed journals. The main eligibility criteria were studies that (1) examined a practically implemented syndromic surveillance system with cluster detection mechanisms, including over-the-counter medication, school and work absenteeism, and disease surveillance relating to the presymptomatic stage; and (2) focused on surveillance of infectious diseases. We identified relevant articles using the title, keywords, and abstracts as a preliminary filter with the inclusion criteria, and then conducted a full-text review of the relevant articles. We then developed a framework for cluster detection mechanisms for various syndromic surveillance systems based on the review.

**Results:**

The search identified a total of 5936 articles. Removal of duplicates resulted in 5839 articles. After an initial review of the titles, we excluded 4165 articles, with 1674 remaining. Reading of abstracts and keywords eliminated 1549 further records. An in-depth assessment of the remaining 125 articles resulted in a total of 27 articles for inclusion in the review. The result indicated that various clustering and aberration detection algorithms have been empirically implemented or assessed with real data and tested. Based on the findings of the review, we subsequently developed a framework to include data processing, clustering and aberration detection, visualization, and alerts and alarms.

**Conclusions:**

The review identified various algorithms that have been practically implemented and tested. These results might foster the development of effective and efficient cluster detection mechanisms in empirical syndromic surveillance systems relating to a broad spectrum of space, time, or space-time.

## Introduction

### Background

Late detection of disease outbreaks has long been a threat to global health security, costing the world many lives, resources, fear, and panic. Case-fatality rates of pandemic diseases are still rising, the most recent being Ebola virus disease in Liberia, West Africa, the Democratic Republic of the Congo, and Uganda [1]. Apart from global fear and panic, Ebola virus disease caused over 11,000 deaths, with national case-fatality rates of about 70% and local economic losses of US $3 to 4 billion [2,3].

Traditional surveillance systems are mostly passive and rely on laboratory confirmations to detect disease outbreaks. These have been enhanced by syndromic surveillance systems [4], which largely depend on visible signs and symptoms and data sources including emergency department records [5], school absenteeism, work absenteeism, disease reporting systems, and over-the-counter medication sales [6,7]. Nevertheless, the existing syndromic surveillance systems cannot detect the disease outbreak early enough, and their data sources and processes exclude the incubation phase of the infection [7]. Disease outbreaks are mostly detected after the infected person is ill or after the terminal stage, thereby increasing the disease burden.

### Clustering Approach and Outbreak Detection

Generally, outbreaks of infectious or communicable diseases are more likely to present in cluster form either in space, time, or both [8,9]. Clustering methods to detect disease outbreaks help identify environmental factors and spreading patterns linked to certain diseases [10]. This was realized many years ago by John Snow, who observed a correlation between cholera disease and a public water source [11]. Barker et al reviewed the dispersal, persistence, and control of some common viruses in the domestic home and in community facilities and concluded that “there is growing evidence that person-to-person transmission via the hands and contaminated fomites plays a key role in the spread of viral infections” [12].

Clustering approaches can be roughly categorized as temporal, spatial, and spatiotemporal. Spatial clustering uses multidimensional vectors with longitudinal and latitudinal coordinates. There are variety of related algorithms, such as density-based spatial clustering of applications with noise (DBSCAN) [8,9,13]. Temporal clustering deals with data points associated with time [14,15]. It includes various algorithms such as cumulative summation (CUSUM) and considers what is strange about a recent event [16-18]. Spatiotemporal clustering involves a time dimension (temporal information) and space dimension (spatial information) [8,9,13]. There are a variety of strategies, including different distance functions [19,20], importing time to the spatial data, transforming spatiotemporal data to the new objects, progressive clustering, and spatiotemporal pattern discovery [8,13]. Aberration detection is mainly performed through thresholding mechanisms, including various forms such as the number of standard deviations from the mean (*z* score), generalized likelihood ratio, recurrence interval, and confidence intervals [21,22].

### Objectives

There have been notable efforts to bridge the gap between a disease outbreak and its late detection. Research in syndromic surveillance is aimed at detecting disease outbreaks at the presymptomatic stage [7]. One of the main concerns is the choice of reliable algorithms that can be used for empirical implementations. Therefore, our general objective was to systematically review reports of practically implemented disease surveillance algorithms for their usage and performance efficacies, and to develop an efficient cluster detection mechanism framework. The results are targeted at people who need to implement efficient syndromic surveillance systems for applications such as over-the-counter medication, school and work absenteeism, and disease surveillance relating to presymptomatic stages, among others. The scope was to review practically implemented state-of-the-art algorithms relating to temporal, spatial, and spatiotemporal clustering mechanisms. We considered various challenges such as user mobility, privacy and confidentiality, and geographical location estimation.

## Methods

### Inclusion and Exclusion Criteria

We developed the inclusion and exclusion criteria based on the objective of the study and through rigorous discussions among the authors. For an article to be included in the review, the study required the following criteria: (1) a study of a practically implemented syndromic surveillance system with cluster detection mechanisms or that was thoroughly assessed with real data (such studies also contributed to the understanding of how privacy and security-preserving methods could be adopted in related studies), (2) a focus on surveillance of infectious diseases such as influenza, cholera, severe acute respiratory syndrome, and Ebola virus disease, (3) a focus on humans, (4) reported in English, (5) journal articles, conference papers, or presentations.

All searches were done without restriction on time boundaries. We excluded any article outside the above-stated scope.

### Literature Search

We conducted a literature search between January and March 2018 in Google Scholar, ScienceDirect, PubMed, IEEE Xplore, ACM Digital Library, and Scopus. We used keywords such as “spatiotemporal clustering,” “syndromic surveillance,” “real time,” “cell phone,” “mobile phone,” “smart phone,” “trajectory,” “aberration detection,” and “clustering.” To improve the search strategy, we combined keywords using the Boolean operators AND, OR, and NOT. We considered peer-reviewed journals and articles.

Guided by the inclusion and exclusion criteria, we conducted a basic filtering by skimming the titles, abstracts, and keywords to retrieve records that seemed relevant. We removed duplicates and fully read and judged articles that seemed relevant based on the inclusion and exclusion criteria. We retrieved other relevant articles from the reference lists of the accepted articles. We recorded the article selection and screening in a Preferred Reporting Items for Systematic Reviews and Meta-Analyses flow diagram [23].

### Data Collection and Categorization

We developed our data collection and categorization methods based on the objective and through literature reviews and discussions among the authors. We defined the categories exclusively to assess, analyzed, and evaluate study (Table 1) [21,24,25].

**Table 1:** Data categories and their definitions.

Category	Definition
Clustering and aberration detection algorithm	The kind of clustering and aberration detection algorithm used and implemented in the study.
Type of clustering algorithm	The type of algorithm used (spatial, temporal, or spatiotemporal algorithm).
Threshold	The type of threshold used to generate alarms and alerts in the study.
Design method	The design method used in implementing the system, such as prototype, participatory or joint application development, or agile or waterfall model.
Evaluation criteria	The criteria used to evaluate the algorithms.
Performance metrics	The performance metrics used to evaluate the algorithms, such as sensitivity, specificity, and positive predictive value.
Type of location	Locations used in clustering, including geolocation, postal codes, and counties; specifies the exact type of location used in the system.
Source of location	Where the type of location information was obtained.
Nature of location	State of the location as static or dynamic.
Visualization tool	The type of tool used to implement the visualization aspect of the system.
Display report	The type of visual displays (eg, graphs, maps, time series) implemented by the various systems in the study.
Design layout	The stages and processes used in the architectural design of the syndromic surveillance system (eg, a layout may consist of data acquisition, clustering and aberration detection, and visualization [21], or may include privacy-preserving mechanisms, machine learning techniques in processing the data, and other layers [24,25]).

### Literature Evaluation and Analysis

We assessed, analyzed, and evaluated eligible articles based on the above-defined categories. We analyzed each of the categories listed in Table 1 to evaluate the state-of-the-art approaches. We calculated percentages of the attributes of the categories based on the total count of each attribute. Note that some studies used multiple categories; therefore, the counts of these categories could exceed the total number of articles reporting on these systems.

### Framework Development

We used state-of-the-art methods from the review as input to develop a cluster detection mechanism framework for disease surveillance systems, including those relating to emergency department records, school and work absenteeism, over-the-counter drugs, and medication sales.

## Results

### Relevant Articles

**Figure 1:**
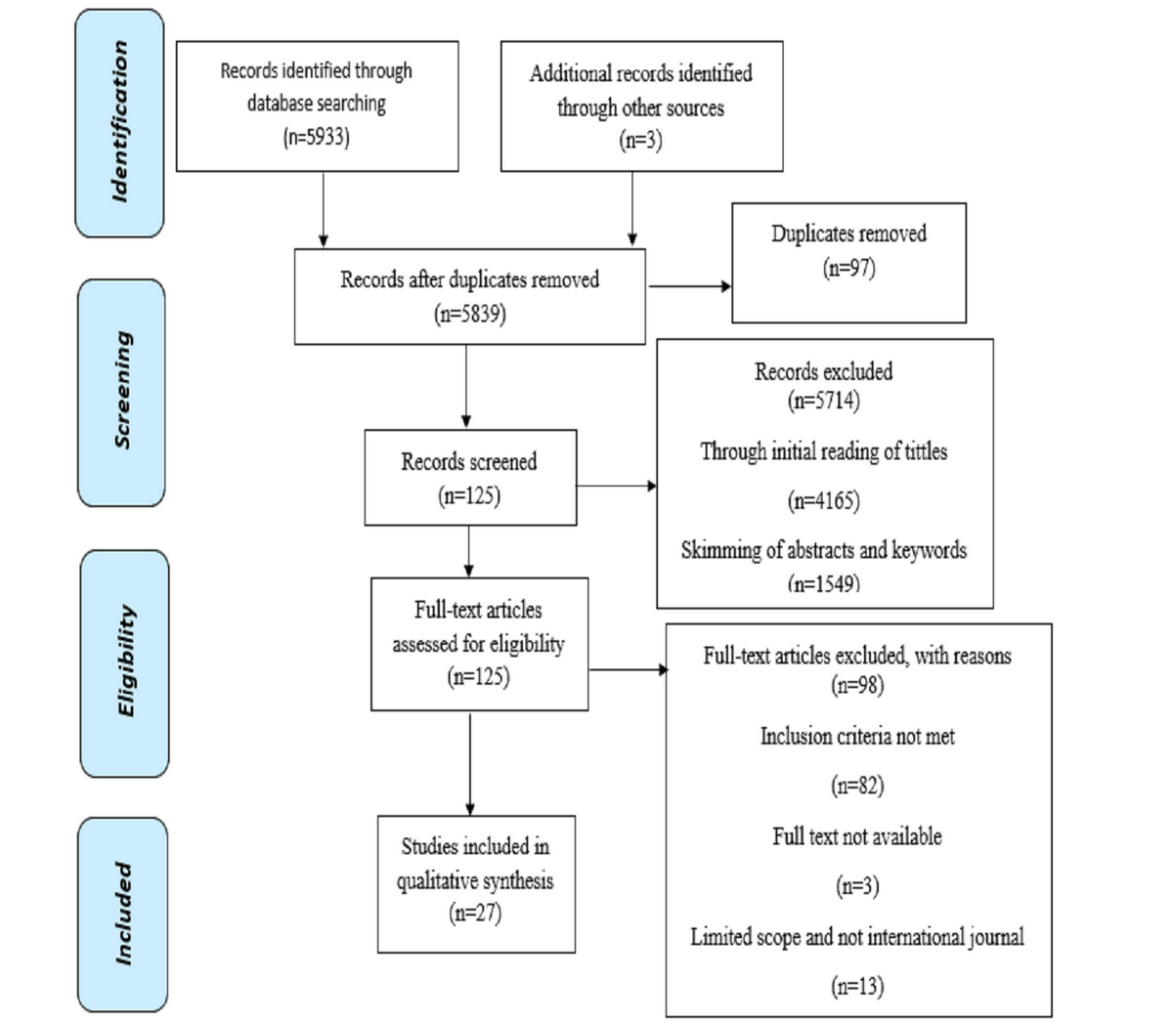
Preferred Reporting Items for Systematic Reviews and Meta-Analyses (PRISMA) flow diagram of the literature review process.

Our search of the various online databases found a total of 5936 records. Removal of 97 duplicates resulted in 5839 records. An initial reading of titles excluded 4165 articles. We excluded a total of 1549 through skimming of abstracts and keywords. An in-depth full-text analysis of the resulting 125 articles, guided by the inclusion and exclusion criteria, excluded 98 articles. Thus, we included a total of 27 articles in the qualitative synthesis (Figure 1).

### Literature Evaluation and Analysis

We assessed, analyzed, and evaluated the 27 articles based on the above-defined categories. The following sections describe the findings.

#### Articles Reviewed

Table 2 [16,21,22,24-47] lists the articles reviewed with their respective targeted diseases, input source, and where and when they were used. Most of the input sources were chief complaints and symptoms reported at the emergency department.

#### Types of Clustering Algorithms

Among the 3 types, namely spatial, temporal, and spatiotemporal, of clustering algorithms, the spatiotemporal algorithm (19/50, 38%) was the most preferred approach, followed by spatial (16/50, 32%) and temporal algorithms (15/50, 30%).

#### Clustering and Aberration Detection Algorithms

A variety of clustering and aberration detection algorithms were implemented in the reviewed articles. Space-time permutation scan statistic (STPSS) and CUSUM algorithms were most widely used, followed by space-time scan statistic and space scan statistic (Table 3).

**Table 2:** Summary of articles reviewed.

Reference (first author, year)	Target disease	Place	Period	Input source
Gesteland, 2003 [26]	Bioterrorism	2002, Olympics	2002	Chief complaints from emergency departments
Yan, 2013 [27]	Infectious diseases	Rural China	2012	Symptoms of patients from health facilities, medication sales from pharmacies, and primary school absenteeism
Maciejewski, 2009 [28]	Detection of public health emergencies	Indiana State Department of Health	2009-2010	Symptoms in emergency departments
Thapen, 2016 [29]	Generalized disease nowcasting	United Kingdom	2014	Twitter and *GP In Hours* weekly bulletin
Thapen, 2016 [30]	Infectious diseases, eg, hay fever and flu	England and Wales	2014	Twitter
Gomide, 2011 [31]	Dengue	Observatório da Dengue website (www.observatorio.inweb.org.br/dengue/)	N/A^a^	Twitter
Qi, 2013 [32]	Influenza infection	University campus	Spring 2011	Movement trajectory
Mathes, 2017 [33]	Infectious diseases	New York City	Since 2001	Emergency department visits with infectious diseases such as cough, sore throat, and fever for influenzalike illness
Yih, 2010 [34]	Acute illness for bioterrorism event	Greater Boston area, Greater Twin Cities area, Austin and Travis County, San Mateo County	2007-2008	Ambulatory care encounters
Kleinman, 2005 [16]	Lower respiratory tract infection	Boston area	N/A	Ambulatory care encounters
Dafni, 2004 [35]	Emergency department data	Athens, 2004 Olympic Games	2002-2003	Symptoms in emergency department
Wagner, 2004 [36]	Infectious disease	Utah, Atlantic City	1999	Chief-complaint data
Weng, 2015 [37]	Enterovirus and influenza	Taipei	2010/2011	School-based syndromes
Maciejewski, 2010 [38]	Respiratory illness	State of Indiana	2007	Infectious disease
Higgs, 2007 [39]	Comprehensive tuberculosis data	San Francisco homeless	1991-2002	Tuberculosis
Ali, 2016 [24]	Infectious disease	Pakistan	2011-2015	Chief complaints from emergency departments
Groeneveld, 2017 [25]	Infectious disease	Netherlands	2014/2015	Respiratory tract infection, hepatitis, and encephalitis/meningitis
Kajita, 2017 [22]	Emergency department data	Los Angeles County Department of Public Health, 2015 Special Olympic Games	2015	Monitor health impact
Choi, 2010 [40]	Infectious disease	Hong Kong	2005	Febrile patients
Heffernan, 2004 [41]	Emergency department chief complaint	New York City Department of Health and Mental Hygiene	2001-2002	Infectious disease, eg, respiratory, fever, diarrhea, and vomiting
Takahashi, 2008 [42]	Infectious disease	Massachusetts	2005	Daily syndromic surveillance data
Besculides, 2005 [43]	Infectious disease	New York City	2001-2002	School absenteeism data
Blake, 2016 [44]	Poliomyelitis outbreaks	N/A	2003-2012	Reporting of acute flaccid paralysis cases and laboratory confirmation
Greene, 2012 [45]	Gastrointestinal disease outbreak detection	Kaiser Permanente Northern California	2009	Data streams from electronic medical records
Vilain, 2016 [46]	Infectious disease	French Institute for Public Health Surveillance, Reunion Island	2013-2014	Emergency department visits
Sharip, 2006 [21]	Infectious disease	Los Angeles County	2003-2004	Emergency department syndromic data
Duangchaemkarn, 2017 [47]	Infectious disease	N/A	2016-2017	Chief complaint symptoms

^a^N/A: not available.

**Table 3:** Frequency of clustering and aberration detection algorithms (n=66).

Algorithm	Usage, n (%)
Cumulative summation	10 (15)
Space-time permutation scan statistic	10 (15)
Space-time scan statistic	5 (8)
Space scan statistic	4 (6)
Kernel density	3 (5)
Moving average	3 (5)
Log-linear regression	2 (3)
Density-based spatial clustering of applications with noise	2 (3)
Recursive least square	2 (3)
Statistical process control	2 (3)
Autoregressive integrated moving average	2 (3)
Risk-adjusted support vector clustering	1 (2)
Bayesian spatial scan statistic	1 (2)
Exponentially weighted moving average	1 (2)
Flexible space-time scan statistic	1 (2)
k-means clustering	1 (2)
K-nearest neighbor with Haversine distance	1 (2)
Shewhart chart	1 (2)
Pulsar method	1 (2)
Risk-adjusted nearest neighbor hierarchical clustering	1 (2)
Small area regression and testing	1 (2)
Spatiotemporal density-based spatial clustering of applications with noise	1 (2)
What is strange about recent event	1 (2)
Bayesian space-time regression	1 (2)
Generalized linear mixed model	1 (2)
Generalized linear model	1 (2)
Holt-Winters exponential smoother	1 (2)
Temporal scan statistic	1 (2)
Modified Early Aberration Reporting System C2	1 (2)
Temporal aberration detection	1 (2)

#### Threshold Detection Mechanisms

An aberration is detected mainly using thresholding mechanisms and, in this regard, various types of approaches were implemented in the reviewed articles. Recurrence interval (10/17, 37%) and *z* score (10/17, 37%) were the most widely used, followed by generalized likelihood ratio (5/17, 18%), confidence interval (1/17, 4%), and incidence ratio (1/17, 4%).

#### Design, Evaluation Methods, and Performance Metrics

The most widely used performance metrics were sensitivity (11/25, 44%) and specificity (9/25, 36%), followed by timeliness (2/25, 8%), and consistency, correlation, and positive predictive value (each 1/25, 4%). The reviewed studies used various evaluation strategies, among which simulation with historical data (12/15, 80%) was the most widely used approach, followed by comparison with known outbreak (2/15, 13%) and power of cluster detection test (1/15, 7%).

**Figure 2:**
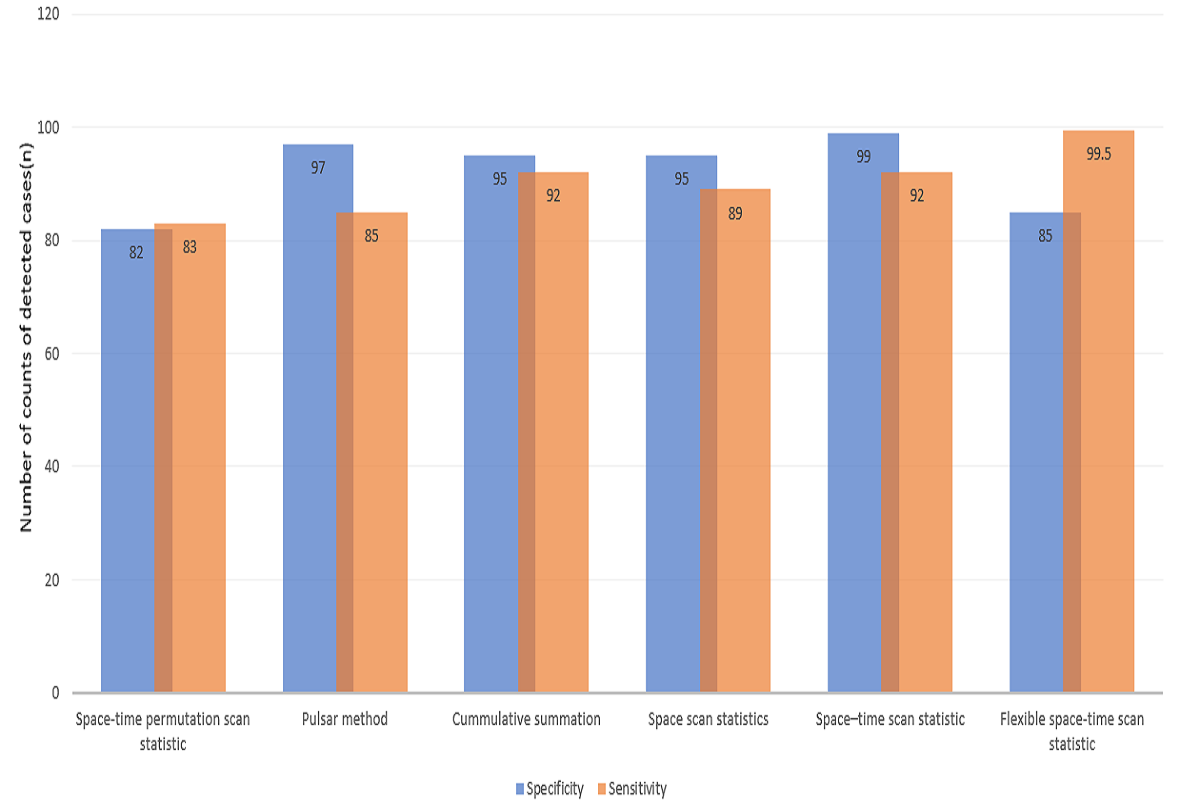
Sensitivity and specificity of the evaluated algorithms.

At specificities and sensitivities ranging from 82% to 99.5%, spatial and spatiotemporal algorithms detected on average more cases (Figure 2, Table 4). Prototype and participatory design were used in the studies. Of 5 systems that disclosed their design methods, 4 used a participatory approach.

**Table 4:** Evaluation metrics of some algorithms.

Algorithms	Specificity (%)	Sensitivity (%)	Detected cases (n)
Space-time permutation scan statistic	82	83	26
Pulsar method	97	85	223
Cumulative summation	95	92	212
Space scan statistic	95	89	790
Space-time scan statistic	99	92	3
Flexible space-time scan statistic	82	99.5	4

#### Location Type and Nature, and Source of Location

The studies used a variety of location type, nature, and source. The majority of studies used static location (22/26, 79%) and the rest used a dynamic location (6/26, 21%). The studies used various address: geocode (14/37, 50%), zip code (13/37, 46%), and county (1/37, 4%). Various sources of locations were used: patient health record (18/27, 64%), mobile device (4/27, 14%), Transport Control Protocol/Internet Protocol (3/27, 11%), county (1/27, 4%), and school address (1/27, 4%).

#### Visualization Tools and Visual Displays

Clustering and aberration detection mechanisms in disease outbreaks need to be supported by excellent visualization tools and display to facilitate a quick response from the concerned bodies on the exact timing and place. In this regard, the reviewed articles used various kinds of tools: ArcGIS (3/9, 24%), Google Maps (2/9, 22%), Twilio (2/9, 22%), OpenStreetMap (1/9, 11%), and JFreeChart (1/9, 11%) were the most widely used. For displaying mechanisms, a map (14/30, 47%) was the most widely used, followed by time series (7/30, 27%), graphs (8/30, 23%), and color indicators (1/30, 3%).

#### Design Layout

Table 5 lists the design layouts identified in the studies and their frequencies of use. Space scan statistic, which is a spatial algorithm, was also able to detect an average of 790 cases.

### Framework on Cluster Detection Mechanism

**Figure 3:**
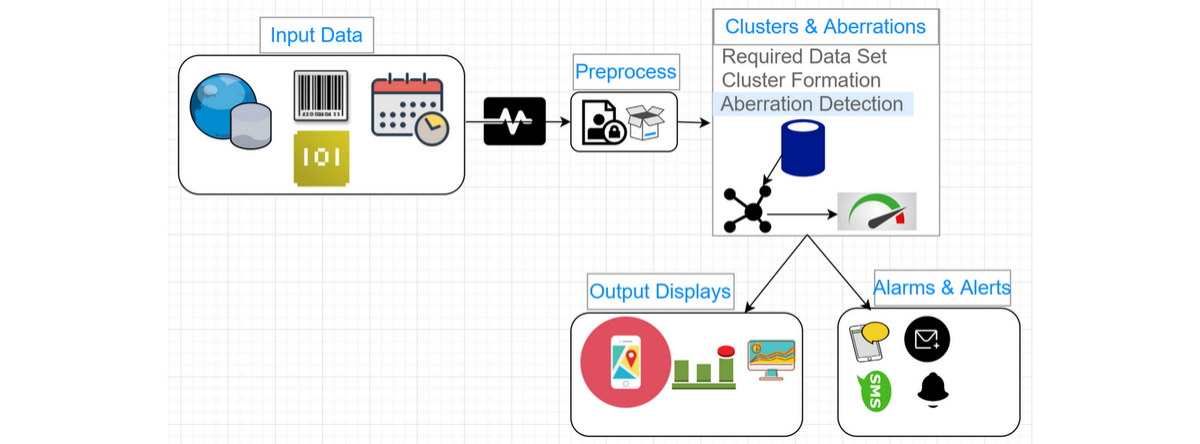
Cluster detection mechanism framework.

We developed a conceptualized framework on cluster detection mechanisms (Figure 3) with input from the principal findings of the systematic review on cluster detection methods. We discuss the various components of the framework below.

**Table 5:** Design layouts and their frequencies (n=22).

Design layout	Description	Usage, n (%)
Data clustering and aberration detection, alarms and alerts (DCADAA)	This layout consists of obtaining data first. Then clustering and aberration detection are done, followed by generating alarms to create alerts of aberrations [16].	12 (55)
Data clustering and aberration detection, visualization, alarms and alerts (DCAVAA)	A visualizing module is built in addition to processes defined in DCADAA [24].	1 (5)
Data cleaning and transformation, clustering and aberration detection visualization, alarms and alerts	In addition to the DCAVAA layer, this layer has data cleaning and transformation features.	3 (14)
Data clustering, filtering or categorizing, aberration detection, alarms and alerts	In addition to DCADAA, this layout filters data or categorizes the data into some defined groups, either manually or by employing machine learning techniques.	2 (9)
Data clustering and aberration detection, privacy-preserving mechanism (DPVCAAA)	In addition to DCAVAA, this layout has privacy-preserving mechanisms, such as anonymization and pseudonymization [27,48].	2 (9)
Real time, privacy-preserving mechanism, data clustering and aberration detection, alerts and alarms	On top of the DPVCAAA layout, there is an additional module for real-time data processing [24,48].	1 (5)
User tracking, data clustering, aberration detection, visualization, alarms and alerts	In addition to DCAVAA, this layout tracks the user’s movement to obtain data. This is followed by validating the data before clustering and aberration detection [24,25].	1 (5)

#### Input Data

Generally, syndromic surveillance systems require input data varying from structured to semistructured data such as comma-separated values, xml, or JavaScript Object Notation (JSON) formats (Figure 3). Ultimately, some key data input elements are highly required for these algorithms. These data elements include the data points with their associated geolocations, date, and time of occurrences [47]. The data points would also have unique nonpersonal identifications and would be associated with their corresponding date, time, and geolocation of occurrences. The data could be in a certain format such as xml, which can be accessed online.

#### Preprocessing Phase

The preprocessing phase is to ensure that the input data is in the right format for the cluster and aberration detection phase to use. Therefore, the framework provides for data conversion. For instance, online data in xml format can be converted to JSON format. Missing data would also be handled in various ways. In most instances, missing data were excluded from the analysis [29]. This and other methods would be used.

Another provision is to ensure that privacy-preserving mechanisms are in place. This framework has a provision in the data preprocessing section to ensure that the input data are devoid of personal data. This would be done by following layout standards and regulations such as the General Data Protection Regulation established by the European Union [48,49]. According to Data are considered nonpersonal if pseudonymization and anonymization methods of privacy-preserving mechanisms are used [50]. Such techniques mitigate risk and assist the data processors in meeting their data compliance requirement. Pseudonymization replaces the most identifying fields within a data record with artificial identifiers or pseudonyms, but it does not replace all personal identifiable information from the data. It basically reduces the linkage of a dataset with the original identity of an individual. Pseudonymization methods use techniques including encryption schemes. With anonymization, a variety of methods are available, and the choice will depend on the degree of risk and the intended use of the data. Some of the methods are direct replacement, scramble, masking, and blurring.

#### Cluster and Aberration Detection Phase

The heart and brain of this framework is the cluster and aberration detection phase. In this layout, clusters and aberrations would be detected by considering the clustering and aberration detection algorithms found in the review. STPSS is very outstanding, since it does not require population-at-risk data to draw the expected baseline value. Rather, it uses the detected cases to determine the expected count [51]. This approach provides significant trend-of-baseline data while avoiding inclusion of historical data that is irrelevant to the current period.

#### Visualization, Alert, and Alarms

The main output of the framework is timely alerts through alarms and visualizations of detected aberrations. In the studies, various visualization tools and output displays were used. Guided by the results and discussion sections of this review, ArcGIS or Google Maps can be used to implement the visualization module. This visual display would mainly be a map with other displays such as a time series and graph. The maps would indicate where and when clustering and aberrations occur. Also, alerts would be triggered through alarms and messaging.

## Discussion

### Overview

The general objective of this study was to systematically review practically implemented disease surveillance algorithms for their usage and performance efficacies and to develop an efficient cluster detection mechanism framework. The results were targeted at individuals and organizations who want to implement efficient syndromic surveillance systems for applications such as over-the-counter medication, school and work absenteeism, and disease surveillance relating to presymptomatic stages, among others. The scope was to review the practically implemented state-of-the-art algorithms relating to temporal, spatial, and spatiotemporal clustering mechanisms. We proposed a framework based on the results of the review and considered various challenges, such as user mobility, privacy and confidentiality, and geographical location estimation. In exploring suitable algorithms, we included in the review studies that assessed syndromic surveillance systems with real data. In addition to thoroughly assessing these algorithms, such studies also contributed to the understanding of how privacy- and security-preserving methods could be adopted in related studies. This is also very important in this field, since personal data need to be handled properly in related studies to preserve security and privacy. For instance, in a related study [16], a privacy agreement with the health plan that provided the data required the researchers to use the exact locations only to get the grouped data.

### Principal Findings

Table 6 summarizes the principal findings of the review. Below, we discuss the algorithms and other dimensions of the findings.

**Table 6:** Summary of the most used categories.

Category	Most used
Clustering algorithm	Space-time permutation scan statistic
Type of clustering	Spatiotemporal type
Threshold	Recurrence interval
Design method	Participatory design
Evaluation method	Simulation with historical data
Performance metric	Sensitivity
Type of location	Geocode
Source of location	Patient health record
Nature of location source	Static
Visualization tool used	ArcGIS
Displayed output	Maps
Layout	Data clustering and aberration detection, alarms and alerts

#### Spatiotemporal Methods

The review identified various spatiotemporal algorithms used for disease surveillance systems, including STPSS, space-time scan statistic, generalized linear mixed model, Bayesian space-time regression, and flexible space-time scan statistic. Spatiotemporal methods generally aimed at detecting disease outbreaks in both spatial and temporal patterns.

STPSS, which was used in many of the studies, was developed to detect hot spots of space-time interaction within space and time pattern occurrences of diseases [52]. Space and time of potential disease outbreak detection is a very efficient method, since health management services can plan for potential outbreaks, knowing where and when to allocate resources to potential outbreak areas. Another reason for its high usage count could be that the algorithm does not require data on the population at risk to draw the expected baseline value, but rather dwells on the detected cases to determine the expected count [51]. This approach provides a significant trend-of-baseline data while avoiding inclusion of historical data that is irrelevant to the current period. STPSS, unlike most of the algorithms, does not draw its baseline data (expected cases) from inaccurate population-at-risk, a control group, or other data that provide information about the geographical and temporal distribution of the underlying population at risk. Such baseline data are inaccurate because there is significant geographical variation in health care utilization data due to differences in disease prevalence, health care access, and consumer behavior [51]. Because of its popularity, Malizia evaluated STPSS for its efficiency and deemed it to be accurate [52].

On the other hand, STPSS is more accurate when used for outbreaks that start locally [51]. Chen et al, who studied spatial and temporal aberration detection methods for disease outbreaks in syndromic surveillance systems, observed that spatial scan methods only detect clusters in basic regular shapes such as cylindrical, circular, or spherical [18]. The spatial scan algorithm does not also consider prior knowledge such as the impact of the infection rate, or size or shape of the outbreak, and it is computationally expensive, as local cluster search requires searching over a large geographical region. These suggest that STPSS is not suitable for detecting disease outbreaks that occur simultaneously in the entire surveillance area. For instance, disease outbreaks that occur through exposure to an infectious agent implies that infected people might be living in different neighborhood. Thus, STPSS will not detect disease outbreaks with very few cases, such as 1 case of smallpox or 3 cases of anthrax in the anthrax bioterrorism that occurred in 2001 [51]. STPSS is only efficient on disease outbreaks with a higher rate of early symptoms [51]. An evaluation using syndromic surveillance data spiked with simulated injections revealed low detection in the spatial and spaciotemporal algorithms [33]. For instance, in an evaluation exercise, at a specificity of 95%, the STPSS detected none [33]. This was due to the geographically disaggregated data, which resulted in a loss of power of detection by the STPSS algorithm [33]. Syndromic surveillance systems are optimally effective when both spatial and temporal cluster detection methods work in unison to track emerging infectious diseases at an early stage over the surveillance area [18,53].

#### Spatial Methods

The spatial methods we identified in this review were space scan statistic, kernel density, Bayesian spatial scan statistic, k-means clustering, DBSCAN, and K-nearest neighbor (K-NN). Unlike spatiotemporal algorithms, spatial algorithms basically concentrate on where aberrations would occur. This makes planning difficult for health management, since it is difficult to know when to implement health interventions, if potential outbreak areas are known. Thus, spatial algorithms are suggested to be implemented together with temporal algorithms [47] to give the surveillance system spatiotemporal properties. According to Duangchaemkarn et al, who evaluated symptom-based data preprocessing for the detection of disease outbreaks with time series and the K-NN algorithm [47], K-NN algorithms potentially are an efficient method for syndromic surveillance; they suggested that the algorithm be further assessed with temporal methods. K-NN and CUSUM were also statistically assessed to be feasible for analyzing nearest neighbor statistics [54]. In such a combined approach of spatial and temporal methods, K-NN would provide clustering patterns of disease occurrences and CUSUM would provide the temporal aspect. CUSUM can spot an aberration in the surveillance area with the mean distances of emerging diseases of various points in the surveillance area [53,54]. Kulldorff et al also supported this opinion by emphasizing that “efficient disease surveillance will need the parallel use of different methods, each with their own strengths and weaknesses” [51]. A syndromic surveillance system is optimally effective when both spatial and temporal cluster detection methods work in unison to track emerging infectious diseases at an early stage over the surveillance area [18,53].

#### Temporal Methods

As Table 3 shows, temporal methods found in the study were CUSUM, moving average, recursive least square, autoregressive integrated moving average, pulsar method, temporal scan statistic, temporal aberration detection, and small area regression and testing. Among these methods, CUSUM was the most commonly used temporal algorithm in our review.

CUSUM is a statistical control method that has traditionally been used for industrial process control. It has been predominantly used in tracking changes in average production process levels since the 1950s [55,56]. The main role of CUSUM in production control is to generate an alert if products from a production process do not conform to defined limits [57]. CUSUM has also been found to be very useful in electronic disease surveillance. The CUSUM algorithm accumulates the variances between detected or observed cases and baseline values over a given time [53,55]. If the CUSUM value is greater than the baseline by a specified threshold, a likelihood aberration is detected [55]. In disease surveillance, CUSUM has been demonstrated to be a very sensitive, fast-reactive method of detecting disease outbreaks and to generate fewer false-positive alarms than more conventional methods [44,55,58]. CUSUM is also among the most commonly used temporal algorithms due to its powerful and straightforward design and implementation [59]. An evaluation study comparing the autoregressive integrated moving average, temporal aberration detection, CUSUM, and Pulsar methods showed temporal aberration detection to be more timely in some syndromes, further empirical assessments in varying datasets are required to conclude which are the best methods [35].

#### Thresholding

The most used threshold for aberration detection in spatiotemporal algorithms was the recurrence interval, possibly as a result of the combination of recurrence interval and Monte Carlo replication, which helps to easily determine and set the specificity of the system [42]. The Monte Carlo simulation is a probability module that is often used with the recurrence interval in clusters to draw a threshold and to determine the likelihood occurrence of a cluster by chance within a specified period for which the analysis is repeated in a regular basis. For instance, in a daily analysis, if the Monte Carlo replication is set to 999 with a statistical significance of *P*<.001, the recurrence interval would be 1000 days, since in disease surveillance the recurrence interval is the inverse of the *P* value [42]. This implies that, for each 1000 days, the expectation of false alarms would be an average of 1 false signal per 1000 days, or 2.7 years, and the recurrence interval would be set to the number of days of the baseline data [34]. The significance level of *P*<.001 is the probability of accepting the occurrence of a cluster by chance within a specified period.

In the reviewed studies, CUSUM is a temporal algorithm that was mostly used together with special algorithms to form spatiotemporal algorithms [60]. Its ease of use and efficiency might have accounted for the high usage [60]. About 60% of the algorithms were classified in the threshold-based category [8]. This corresponded to relatively high usage of spatiotemporal algorithms. Most of these algorithms employed cylindrical risk regions to detect clusters. The radius formed the area of the map, while the height represented the time. The radius and time were varied to some upper bound thresholds.

#### Design and Evaluation

Participatory design was mostly used at the design stage, while simulation with historical data was mostly used to evaluate the clusters in most of the algorithms. Historical data were mostly used perhaps because those records were known to have aberrations, making it possible and easy to determine the performance of the system. Sensitivity and specificity were the most used performance metrics in the evaluation. This could be because users wanted a system with reduced false-alarm rates.

Some of the algorithms were compared based on their performance metrics of sensitivity, specificity, timeliness, and positive predictive value (Figure 2, Table 4) [33,61]. Considering Table 4 and Figure 2, at an average sensitivity and specificity of 82%, STPSS detected more cases (n=26). At a very high sensitivity and specificity up to 99.5%, the special and spatiotemporal algorithms continued to detect high numbers of cases. At a slightly lower sensitivity and specificity ranging from 82% to 92%, the temporal algorithms also detected some cases. In using spatiotemporal clustering algorithms in syndromic surveillance, various methods such as temporal methods and near neighbors should be considered. These measures may augment for the sparseness of data, which could result in a loss of power to detect areas with local excess aberrations in spatial and spatiotemporal methods [44,58].

An evaluation that was performed through injection of spikes of a known outbreak revealed low detection in the space and spaciotemporal algorithms [33,44,58,61]. Space scan statistic detected 3% of all injections, but STPSS detected none at a specificity of 95% [33]. However, the temporal algorithms detected higher percentages ranging from about 2% to 19% of the injections under the same level of sensitivity [33,58,61]. The low detection rates of the spatial and spatiotemporal algorithms could have been because the algorithms were not adjusted to increase their power of detection when applied to disaggregated data [33,44,58,61]. Also, the performance of the algorithms could be enhanced with a higher number of input cases and better coverage in spatial and spatiotemporal algorithms [34].

In terms of location, geocodes of census tracking or hospitals and zip codes were mostly used as location points for the clustering algorithms. These data were mostly retrieved from patient health records. The dynamic nature of the sources of location caused a low count, which could have been because they have not been comparatively assessed and due to difficulties associated with acquiring and processing the dynamic nature of location source data for syndromic surveillance. Privacy-preserving polices and a high computational time requirement prohibited the use of exact location of persons for syndromic surveillance. Exact locations such as house numbers and tracking of individuals were mostly used for group data at the zip code or county level. Information on the exact place of infection is also vital for early prevention and control of morbidity and mortality. But these limitations often hamper the accuracy of information on place of infection, since the information collected often relates to the place of notification, which is usually far from the place of infection [32,48,62]. Also, systems that provided text space for users to indicate their location had some limitations. Users did not indicate proper locations or addresses, so their locations could not be geocoded. This resulted in limited sample sizes [27,29].

#### Visualization and Alerting

ArcGIS was mostly used to display graphs in the studies in this review. It is possible that maps were the most common display type because they can be used to represent both spatial and spatiotemporal data. This could have accounted for their high usage of 34% and 47% in their respective categories. In the system design layout category, most of the systems obtained data from various sources first. Clustering and aberration detection were done, followed by generating alarms to create alerts of aberrations. Tracking for data, acquiring data in real time, privacy-preserving mechanisms, filtering, and data cleaning were some of the layout processes employed in a few of the systems studied. The low rate of tracking persons for data sources could be due to legal, privacy, and ethical reasons [48]. The low count of filtering and data cleaning could be due to implementation challenges, as machine learning algorithms and natural language processing tools are used for effectiveness [32,48,62].

### Conclusion

Despite the numerous availabilities of disease surveillance algorithms, their lack of efficacy in detecting disease outbreaks remains a threat to global health security. To overcome this problem, the main objective of this study was to systematically review practically implemented disease surveillance algorithms for their usage and performance efficacies, and to develop an efficient framework. The results were targeted at individuals and organizations who wish to implement efficient syndromic surveillance systems in applications such as over-the-counter medication, school and work absenteeism, and disease surveillance relating to presymptomatic stage, among others. The scope was to review the practically implemented state-of-the-art algorithms relating to temporal, spatial, and spatiotemporal clustering mechanisms. We considered various challenges such as user mobility, privacy and confidentiality, and geographical location estimation.

The study revealed that STPSS and CUSUM were the most frequently implemented algorithms. These algorithms can be used in syndromic surveillance systems that are aimed at implementing state-of-the-art cluster detection mechanisms, although STPSS was shown to be efficient only in a surveillance system with a high rate of infections. Temporal and spatial algorithms such as CUSUM and K-NN can also be combined in an empirical study to achieve efficient results. This study provided wide data categorization, ranging from design of the system to the display of reports which we used in the development of the framework. These results might foster the development of effective and efficient cluster detection mechanisms in empirical syndromic surveillance systems relating to a broad spectrum of space, time, or space-time.

## Abbreviations

CUSUM: cumulative summation
DBSCAN: density-based spatial clustering of applications with noise
JSON: JavaScript Object Notation
K-NN: K-nearest neighbor
STPSS: space-time permutation scan statistic
